# Molecular epidemiological and genetic variability of *Salmonella* isolates from the tropical mountainous region of southern China (2017–2024)

**DOI:** 10.1128/spectrum.02676-25

**Published:** 2026-03-02

**Authors:** Lebin Su, Haoyuan Lun, Nanyang Li, Xiachen Xian, Baisheng Li, Yingyu Liang, Abdurrahman Hassan Jibril, Minting Jian, Guangxian Xu, Guohua Huang, Zhe Liu, Kaisong Huang

**Affiliations:** 1 Zhaoqing Center for Disease Control and Prevention, Zhaoqing, China; 2 Guangdong Provincial Key Laboratory of Medical Immunology and Molecular Diagnostics, School of Medical Technology, Guangdong Medical University, Dongguan, China; 3 The First People’s Hospital Of Zhaoqing, Zhaoqing, China; 4 Guangdong Provincial Center for Disease Control and Prevention, Guangzhou, China; 5 Duanzhou District Center for Disease Control and Prevention, Zhaoqing, China; 6 Center for Advanced Medical Research and Training, Usmanu Danfodiyo University 108011, Sokoto, Nigeria; 7 Guangdong Provincial Institute of Public Health 652472 , Guangzhou, China; University of Maryland at College Park, College Park, Maryland, USA

**Keywords:** *Salmonella enterica*, foodborne diseases, whole-genome sequencing, antimicrobial resistance

## Abstract

*Salmonella enterica* is a leading foodborne pathogen worldwide, posing significant public health concerns. However, its prevalence in mountainous regions in China remains unclear. This study analyzed 384 *Salmonella* strains isolated from 2,540 samples collected between 2017 and 2024 from humans, food, and environmental sources in tropical mountainous areas of China. Whole-genome sequencing and antimicrobial-susceptibility testing were performed to investigate genomic characteristics and antimicrobial resistance profiles. The results showed that *S*. I 1,4 [5],12:i:- (ST34, 43.5%) was the predominant serovar among diarrheal patients, while *S*. Enteritidis (ST11, 40%) was the primary causative serovar associated with systemic infections characterized by bacteremia. In food and environmental samples, *S*. Typhimurium (ST19, 39.1%) and *S*. Enteritidis (ST11, 35.1%) were most frequently isolated, respectively. Antimicrobial susceptibility testing revealed high resistance rates to ampicillin (63.8%), tetracycline (62.7%), ciprofloxacin (17.7%), cefotaxime (16.4%), and ceftazidime (15.6%), with 115 isolates (29.9%) identified as multidrug-resistant. Notably, six isolates were tigecycline-resistant, including two harboring *tet(X4*) genes on an IncFIA(HI1)-IncHI1A- IncHI1B(R27) hybrid plasmid. The distribution of virulence genes varied across strains from different sources and serovars. Genes encoding carbonic anhydrase (*mig-5*), plasmid-encoded fimbriae (*pefA*, *pefB*, *pefC*, and *pefD*), and type III secretion system effectors (*spvB*, *spvC*, and *spvD*) were significantly more prevalent in strains from patients with systemic infection than in those from diarrhea cases. The *cdtB* gene encoding typhoid toxin was identified in 19 non-typhoidal strains. This study presents the first comprehensive report on the prevalence and genetic dynamics of S. *enterica* in tropical mountainous regions of southern China, offering insights into novel interventions.

**IMPORTANCE:**

*Salmonella* is a serious zoonotic foodborne pathogen and the third leading cause of death from human diarrheal diseases worldwide. In this study, we report, for the first time, a systematic surveillance of *Salmonella* prevalence among clinical patients, food, and environmental sources in the tropical mountainous regions of southern China from 2017 to 2024. Our findings illuminate the prevalence and genetic dynamics of *S. enterica* across diverse sources, as well as differences between patients with diarrheal disease and those with systemic infections characterized by bacteremia. Moreover, we observed high resistance to clinically important treatment drugs, including fluoroquinolones and third-generation cephalosporins, in these regions. These results provide critical evidence to inform more effective surveillance, treatment, and control strategies in tropical settings and underscore the need for continuous, real-time monitoring of pathogen dynamics.

## INTRODUCTION

Foodborne disease (FBD) is a significant public health concern worldwide, causing 600 million cases of illness and more than 420,000 human deaths annually (1). The most common foodborne pathogens are *Campylobacter* spp., *Cryptosporidium* spp., enterotoxigenic and enteropathogenic *Escherichia coli*, norovirus, *Salmonella enterica*, *Listeria monocytogenes*, and *Shigella spp.,* and so forth (2). Among them, *Salmonella* was recognized as one of the four key causes of human diarrheal diseases. Salmonellosis is the third leading cause of death among diarrheal diseases globally (3–5). *Salmonella* is a gram-negative bacterium belonging to the Enterobacteriaceae family and consists of two species: *Salmonella enterica* (*S. enterica*) and *Salmonella bongori* (*S. bongori*). *S. enterica* is further taxonomically classified into six subspecies (subsp), including *S. enterica* subsp. salamae, enterica, arizonae, diarizonae, houtenae, and indica (6). The *S. enterica* subsp. enterica primarily infects avian and mammal animals, while the other *Salmonella* subspecies are mainly found in reptiles and cold-blooded animals (7). Based on the variations of somatic O, flagella H, and Vi-antigen, *Salmonella* is divided into more than 2,600 serovars clustering into around 50 serogroups, in which approximately 1,600 serovars belong to *S*. enterica subspecies enterica (8–10).

*Salmonella* serovars were also commonly classified into typhoidal and non-typhoidal serovars (NTSs) based on their host adaptation (11). Non-typhoidal *Salmonella* (NTS) is a zoonotic foodborne pathogen primarily transmitted through the consumption of contaminated food products such as meat, eggs, milk, fruits, and vegetables. Globally, NTS is responsible for an estimated 180 million diarrheal cases and 298,000 deaths each year, accounting for 41% of diarrheal disease-related deaths (4, 12). While NTS generally causes self-limiting gastroenteritis in healthy adults, invasive NTS (iNTS) can disseminate to the bloodstream, lymph nodes, and other organs, leading to sepsis and life-threatening complications, particularly among young children, the elderly, and immunocompromised individuals (13, 14). Conventionally, the clinical treatment of severe salmonellosis typically involves the empirical prescription of fluoroquinolones and third-generation cephalosporins, or the adoption of treatment strategies based on the local epidemiological situation prior to comprehensive drug susceptibility testing. However, resistance to key antibiotics, especially extended-spectrum cephalosporins, carbapenems, and fluoroquinolones, has been reported in both humans and food animals in recent years (15, 16). Numerous investigations have found a direct link between antibiotic misuse in food animals and the rise of antimicrobial resistance (AMR) in foodborne bacteria linked to human illness (15, 17). Therefore, a better understanding of the prevalence, AMR patterns, and genetic characteristics of locally prevalent *Salmonella* strains is invaluable for developing more effective diagnostics and therapeutic strategies, as well as reducing the disease burden.

Traditionally, researchers used serological agglutination, broth microdilution, and PCR methods to monitor *Salmonella* serovars, antibiotic resistance profiles, and virulence genes. Moreover, pulsed-field gel electrophoresis and multilocus sequence typing (MLST) were employed to investigate the genetic relatedness among the isolated strains. However, these traditional typing methods offer limited resolution in distinguishing closely related isolates, whereas whole-genome sequencing (WGS) provides a more robust and high-resolution approach for comprehensive genomic characterization (18). WGS not only provides detailed insights into virulence and AMR genes but also enables precise assessment of strain relatedness and retrospective comparisons through core/whole-genome MLST (cg/wgMLST) (18, 19). Consequently, WGS-based analysis is becoming the most potent tool for monitoring foodborne pathogens, identifying epidemic sources, and supporting risk assessments worldwide, including for *Salmonella enterica* (19–21).

In China, it has been reported that approximately 70%–80% of FBD outbreaks are attributed to *S. enterica*, posing significant public health challenges (5, 22, 23). The most frequently isolated *S. enterica* serovars associated with human infections in China are *S*. Typhimurium, its monophasic variant (*S*. 4,[5],12:i-), *S*. Enteritidis, and *S*. Derby (22, 24–26). Various other serovars, such as *S*. London, *S*. Rissen, *S*. Corvallis, *S*. Meleagridis, *S*. Kentucky, and *S*. Goldcoast, have also shown increasing prevalence in recent years (24–26). In the coastal, developed cities of South China, particularly in the first-tier city of Shenzhen, *S*. Typhimurium and its monophasic variant (S. 4,[5],12:i-) have emerged as the predominant serovars responsible for human salmonellosis. The prevalence of these multidrug-resistant (MDR) strains is on the rise, as evidenced by systematic surveillance and genomic monitoring utilizing WGS data analysis (27, 28).

Despite increasing data from coastal cities, a significant knowledge gap persists regarding the epidemiological characteristics, AMR patterns, and genomic features of *Salmonella enterica* in the mountainous regions of South China. Zhaoqing, located in Guangdong province, serves as a transitional hub between coastal and inland China, yet has received little research attention. To address this knowledge gap, we conducted a comprehensive retrospective analysis of 384 *Salmonella* isolates obtained from 2,540 samples collected from patients in sentinel hospitals, as well as food and environmental sources in Zhaoqing City, China, between 2017 and 2024. We analyzed both clinical and non-clinical *Salmonella* isolates using WGS to determine serovar diversity, AMR profiles, virulence factors, AMR-associated plasmids, and phylogenetic relatedness. The findings from this study will provide essential knowledge and an updated understanding of the epidemiological and molecular mechanisms underlying *Salmonella* infections in mountainous regions of South China, thereby informing more effective surveillance, treatment, and control strategies.

## MATERIALS AND METHODS

### Sample collection and bacteria isolation

From 1 January 2017 to 31 March 2024, 2,540 samples were collected from diverse sources, including diarrhea patients in sentinel hospitals, food, and the environment, in Zhaoqing City as part of the National *Salmonella* surveillance program by the Zhaoqing Center for Disease Control and Prevention. Food samples include pre-packaged ready-to-eat foods, cooked foods, pastry products, baked goods, pre-prepared dishes, vegetables, etc., while environmental samples were mainly collected from poultry farms, poultry meat processing factories, cooked food processing factories, wastewater from sentinel hospitals, domestic wastewater from sewage treatment plants, etc. Collected samples were initially enriched in TTB/SC broth (Tetrathionate Broth Base, TTB; Selenite Cystine, SC) and then plated on BS (Bismuth Sulfite) agar and XLD (Xylose Lysine Deoxycholate) agar according to the National Food Safety Standards of China document and National Diagnostic criteria for infectious diarrhea (29, 30). Isolates with typical *Salmonella enterica* phenotypes were sub-cultured for purity on XLD plates and further identified using the VITEK 2 compact GN ID cards and matrix-assisted laser desorption ionization-time-of-flight mass spectrometry analysis (Autobio Diagnostics, China). A total of 384 non-replicate *Salmonella enterica* strains were ultimately isolated from 2540 samples, with the detailed information shown in File S1. Clinical and epidemiological data of *Salmonella*-positive patients were retrieved from sentinel hospitals. All participants provided verbal informed consent, and the Research Ethics Committee of the Zhaoqing Center for Disease Control and Prevention approved this study.

### *Salmonella* serotyping

The serotype identification of isolated *Salmonella* strains was performed using the slide agglutination method according to the Kauffmann-White scheme as previously reported (20). Briefly, the grown colonies were first coated on glass slides and then reacted with O and H antigens for agglutination testing following the manufacturer’s instructions (Statens Serum Institut, Denmark). At least five colonies were separately tested for each agar plate.

### Antimicrobial susceptibility testing

Antimicrobial susceptibility of 384 *Salmonella enterica* strains to 14 different drugs was determined using a broth microdilution method according to the Clinical and Laboratory Standards Institute guidelines (31). These 14 drugs include Trimethoprim/Sulfamethoxazole (SXT), Colistin (CT), Ertapenem (ETP), Meropenem (MEN), Cefotaxime (CTX), Ceftazidime (CAZ), Ceftazidime/Avibactam (CZA), Tetracycline (TET), Tigecycline (TIG), Ciprofloxacin (CIP), Azithromycin (AZM), Amikacin (AMI), Ampicillin (AMP), and Ampicillin/Sulbactam (AMS). The *Escherichia coli* strain ATCC 25922 and *Salmonella Typhimurium* strain ATCC 14028 were used as quality control during antimicrobial susceptibility testing. Strains resistant to three or more antimicrobial classes were classified as MDR.

### WGS and bioinformatics analysis

The genomic DNA of 384 *Salmonella enterica* strains was extracted using the Qiagen DNeasy Kit (QIAGEN, China) from the overnight pure culture on LB broth at 37°C, according to the manufacturer’s instructions. Extracted genome DNA quality and integrity were evaluated using the Nanodrop 1000 spectrophotometer and Agilent 2100 Bioanalyzer. The Qubit 2.0 fluorometer was used to quantify the extracted DNA. WGS was performed on either the Illumina NovaSeq 6000 platform (Novogene, China) or the MGISEQ-200RS platform (BGI, China). Raw sequencing read quality was assessed using FastQC v0.11.9, and low-quality sequences and adapter sequences were trimmed using Trimmomatic 0.39 or SOAPnuke 1.5.6 (32, 33). Trimmed clean reads were *de novo* assembled using SPAdes 3.13.0. The assembled genome sequences were further analyzed by MLST and serotype prediction with the Pathogenwatch platform (https://pathogen.watch/). A minimum spanning tree was constructed based on the STs and source of the 384 *Salmonella enterica* isolates using BioNumerics 7.6. Genome annotations were performed using the RAST server (34). Acquired antimicrobial resistance genes (ARGs), plasmid replicons, and virulence genes were identified using the CARD (https://card.mcmaster.ca/home), PlasmidFinder (https://cge.food.dtu.dk/services/PlasmidFinder/), and VFanalyzer in the VFDB database (http://www.mgc.ac.cn/VFs/), respectively (35–37).

In addition, 40 strains containing the resistance genes of *tet(X4*), *Bla_CTX-M-14_*, *Bla*_OXA-1_, *Bla*_LAP-2_, *Bla*_NDM-5_, *Bla*_TEM-244_, and others, and isolates carrying typhoid toxin genes were subjected to nanopore long-read sequencing using the ONT GridION platform (Oxford Nanopore, England). Low-quality sequences and barcode sequences from raw data generated by the ONT GridION were removed using MinKNOW v5.4.0. The Flye 2.9.2 was used to assemble the Nanopore long reads. The generated assembly was further polished using Medaka v1.11.3 with the original nanopore data and corrected using Pilon v1.24 with the Illumina-generated reads. The large plasmid sequence was annotated by the bakta v1.10.2. Visualized comparisons between different plasmids and circular image generation were performed using the BLAST ring image generator (BRIG) v0.95. The heat map of resistance genes was constructed using the ComplexHeatmap 2.21.2 from Bioconductor in R.

### Phylogenetic analysis

The genome SNP analysis of the 384 *S*. *enterica* strains was conducted using the SKA v1.0 program with an optimal Kmer size of 19. Subsequently, a maximum likelihood phylogenetic tree was constructed using FastTree v2 with default parameters based on these SNP alignments. For the 21 isolates carrying the typhoid toxin *cdtB* genes, genome similarity analyses were performed against 16,366 *Salmonella enterica* strains available from the NCBI genome database as of 2024.08.01 using the Fasta ANI v1.32. For each *cdtB*-carrying isolate, the top five strains with the highest genome similarity were selected for further phylogenetic analysis. The phylogenetic tree for these isolates and selected strains was constructed using the SKA and FastTree programs described above. All phylogenetic trees were visualized and annotated using the online iTOL.

### Statistical analysis

All statistical analyses were performed using GraphPad Prism v10.3.1. For comparisons between three or more groups, data were analyzed using the non-parametric Kruskal–Wallis test with Dunn multiple comparison post-test. The two-tailed Mann–Whitney *U* test was utilized for comparisons between two groups.

## RESULTS

### *Salmonella enterica* prevalence and serovar distribution

**Fig 1:**
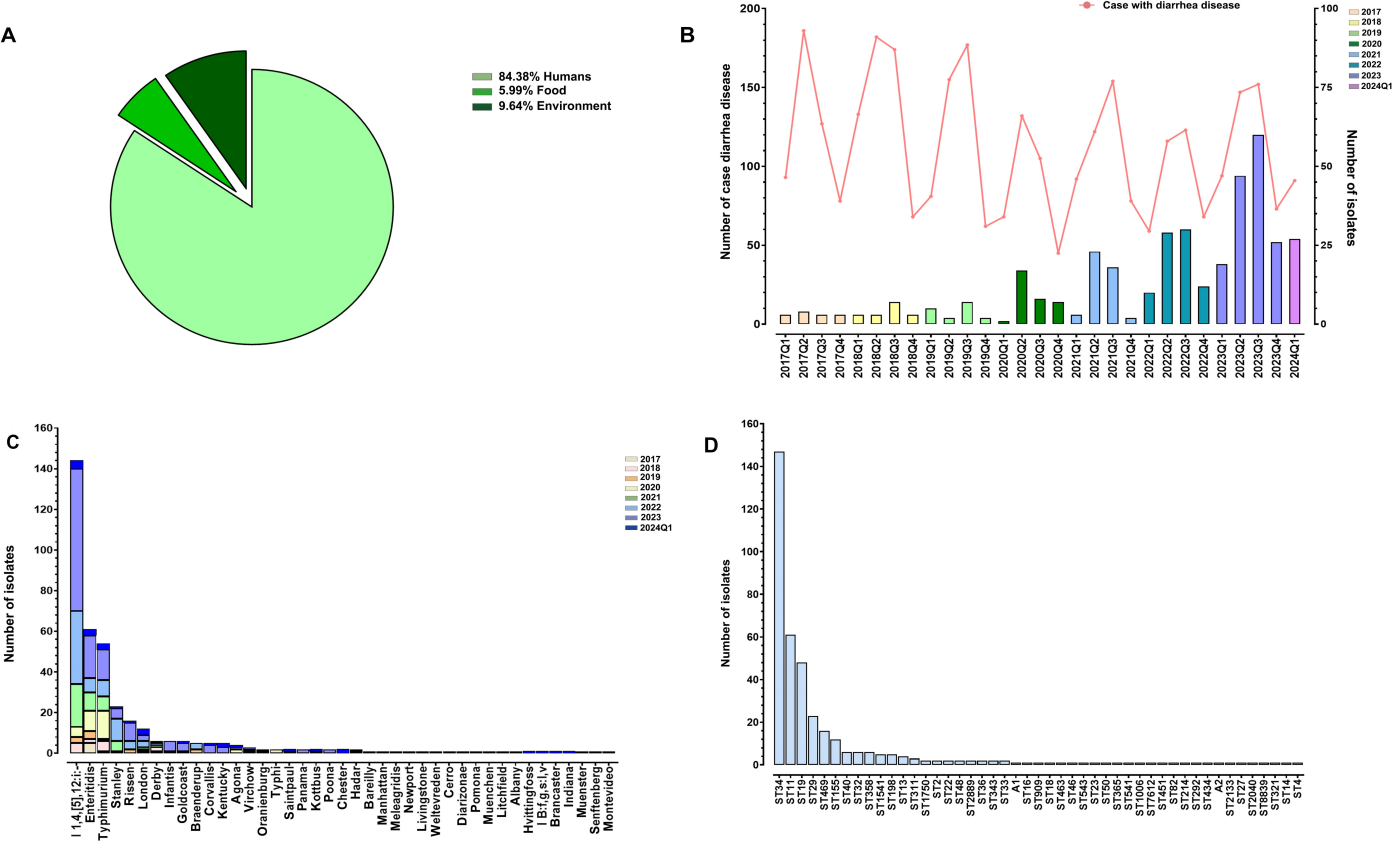
Prevalence and epidemiological characteristics of 384 *Salmonella* strains collected from various sources in Zhaoqing, China, between 2017 and 2024. (**A**) Composition of sources for the 384 *Salmonella* isolates. (**B**) Trends in reported diarrhea cases alongside the number of *Salmonella* isolates across different time intervals from 2017 to 2024. (**C**) Serovar distribution of the 384 *Salmonella* isolates, covering 41 identified serovars. (**D**) Distribution of the 384 isolates across 47 distinct sequence types.

A total of 384 *S*. *enterica* strains were successfully isolated from 2,540 samples collected between 1 January 2017 and 31 March 2024, in Zhaoqing, China. Among these isolates, 324 (84.38%) were recovered from clinical patients, 37 (9.64%) from environmental samples, and 23 isolates (5.99%) from food samples (Fig. 1A). Among the 324 human-derived strains, 289 were isolated from fecal samples, 10 from anal swab samples, 24 from blood samples, and one from a urine sample (File S1). Surveillance data indicated a significant decline in reported diarrhea cases from sentinel hospitals from 2017 to 2024. However, during the same timeframe, there was a notable increase in the number of *Salmonella enterica* isolates obtained (Fig. 1B). Among the 384 isolates, 41 distinct *Salmonella* serotypes were identified using the traditional agglutination testing method. Notably, serotype predictions generated by the Pathogenwatch platform based on genome sequences were 100% consistent with the agglutination results for all 384 isolates. The most prevalent serovar was the monophasic variant of *S*. Typhimurium (*S*. I 1,4,[5],12:i:-), accounting for 37.5% (144/384) of isolates, followed by *S*. Enteritidis (15.9%, 61/384), *S*. Typhimurium (13.8%, 53/384), *S*. Stanley (6.0%, 23/384), and *S*. Rissen (4.2%, 16/384), *S*. London (3.1%, 12/384), *S*. Derby (1.6%, 6/384), *S. Infantis* (1.6%, 6/384)*, S*. Goldcoast (1.6%, 6/384), *S*. Braenderup (1.3%, 5/384), *S*. Corvallis (1.3%, 5/384), *S*. Kentucky (1.3%, 5/384), *S*. Agona (1.0%, 4/384), *S*. Vichow (0.8%, 3/384) (Fig. 1C). A rare *Salmonella enterica* subsp. diarizonae serovar was also isolated and identified from a diarrhea patient. The findings also showed that serovar distribution varied significantly by source. Among the 324 *S*. *enterica* strains isolated from humans, the top five serovars were *S*. I 1,4,[5],12:i:- (43.5%), *S*. Enteritidis (14.2%), *S*. Typhimurium (13%), *S*. Stanley (7.2%), and *S*. Rissen (3.7%). However, of the 324 human-derived strains, 25 strains were isolated from systemic infections (24 from blood and one from urine samples), predominantly consisting of *S*. Enteritidis (40%), *S*. Derby (12%), *S*. Typhimurium (12%), and *S*. Typhi (8%). On the other hand, *S*. Enteritidis (35.1%), *S*. Corvallis (13.5%), *S*. I 1,4,[5],12:i:- (5.4%), *S*. Agona (5.4%), *S*. Chester (5.4%), *S*. Goldcoast (5.4%), *S*. Kentucky (5.4%), *S*. London (5.4%), and *S*. Typhimurium (5.4%) were the top-ranked serovars among the strains recovered from the environmental samples. In contrast, the five most common serovars isolated from food samples were *S*. Typhimurium (39.1%), *S*. Rissen (13.0%), *S*. Agona (8.7%), *S*. Enteritidis (8.7%), and *S*. Hadar (8.7%).

According to the temporal distribution of serovars, a significant rise in the isolation rate of the *S*. I 1,4,[5],12:i:- serovar was observed starting in 2021. Since then, this serovar has accounted for approximately 45% of the total annual isolates across three sources, surpassing *S*. Typhimurium and *S*. Enteritidis and becoming the most predominant serovar. Detailed information on serovar distributions and the epidemiological characteristics of these isolates is available in File S1.

### MLST patterns

**Fig 2:**
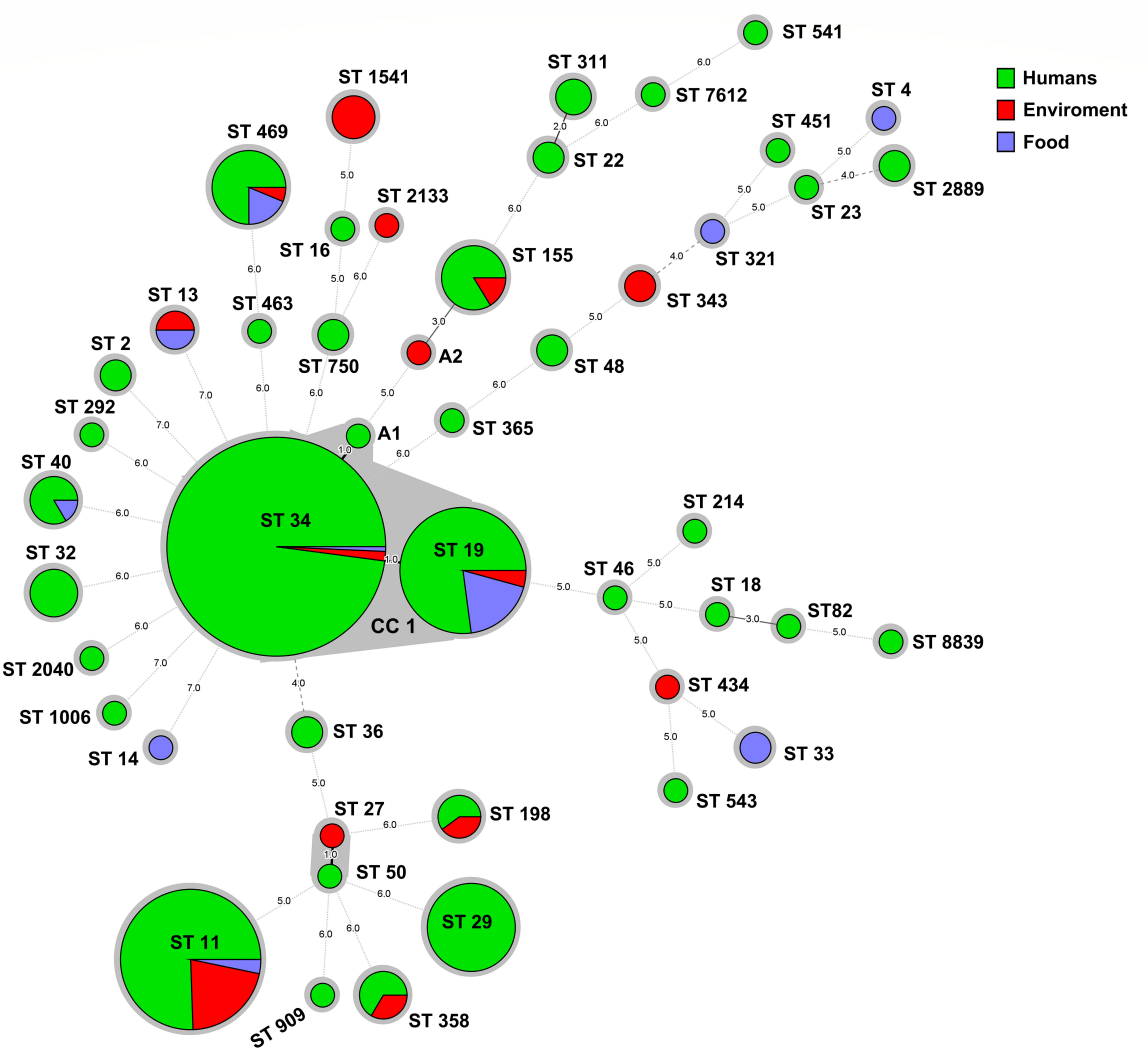
Minimum spanning tree constructed using BioNumerics based on the STs analysis and source of these 384 *Salmonella* strains. Each node represents an ST type, with node size proportional to the number of *Salmonella* strains. The branch length between two nodes reflects the number of allelic gene differences. The color of each node indicates the source of the isolates.

The MLST analysis revealed a total of 47 distinct ST types among the 384 isolates, in which ST34, ST11, ST19, and ST29 were the most prevalent, accounting for 38.28% (147/384), 15.88% (61/384), 12.5% (48/384), and 5.98% (23/384), respectively (Fig. 1D). Of the 147 strains belonging to ST34, 142 strains were serovar of *S*. I 1,4,[5],12:i:- and five were serovar *of S*. Typhimurium. Notably, all 61 *S*. Enteritidis strains belonged to ST11, and all 23 S. Stanley belonged to ST29, while the 48 strains of ST19 were all from the serovar of *S*. Typhimurium. All 16 *S*. Rissen isolates, 12 *S*. London isolates, 6 *S*. Derby isolates, and 6 *S*. Infants isolates were identified as ST469, ST155, ST40, and ST32, respectively. There are two strains (17SAL016 and 24CF1504) with undetermined ST types due to the absence of a hit at the *hisD* locus, which were designated as the A1 and A2 types in this study (Fig. 2). A minimum spanning tree was built using BioNumerics based on the STs and sources of these 384 *Salmonella* strains to further investigate relationships among the various STs of the isolates. The results demonstrated the presence of a predominant clonal cluster CC1 circulating in Zhaoqing, which consists of isolates of ST34, ST19, and A1 types (Fig. 2). Among the top four predominant ST types, ST34, ST19, and ST11 strains were isolated from three different sources: humans, the environment, and food, while the ST29 strains were exclusively isolated from human sources (Fig. 2). The remaining 42 ST-type strains exhibit a polymorphic distribution in the tree (Fig. 2).

### AMR phenotypes, resistome, and plasmidome profiling of *Salmonella* isolates

The antimicrobial susceptibility of the isolated *S. enterica* strains to 14 antimicrobial agents was evaluated using the broth microdilution method. The findings revealed that the highest resistance rates were observed for ampicillin (63.8%, 245/384), followed by tetracycline (62.7%, 241/384), ampicillin/sulbactam (38.8%, 149/384), and trimethoprim/sulfamethoxazole (38.5%, 148/384) (Table 1). However, resistance to ertapenem (0.5%, 2/384), meropenem (0.5%, 2/384), tigecycline (1.3%, 6/384), and ceftazidime/avibactam (1.0%, 4/384) was low. All 384 *Salmonella* isolates were susceptible to colistin. In contrast, resistance to clinically important drugs—ciprofloxacin (17.7%, 68/384), cefotaxime (16.4%, 63/384), and ceftazidime (15.6%, 60/384)—was notably higher (Table 1). Strains resistant to three or more antimicrobial classes were classified as MDR. Among the 384 *S*. *enterica* isolates, 115 strains (29.9%) were identified as MDR, with the most common resistance pattern involving trimethoprim/sulfamethoxazole-tetracycline-ampicillin-ampicillin/sulbactam (File S2). Additionally, 41 isolates demonstrated resistance to cefotaxime-ceftazidime-ampicillin. Of the 115 MDR strains, the serovars *S*. I 1,4,[5],12:i:- and *S*. Typhimurium collectively accounted for 80% (92/115), belonging to sequence types ST34 and ST19, respectively. The *S*. I 1,4,[5],12:i:- MDR isolates were primarily resistant to trimethoprim/sulfamethoxazole, cefotaxime, tetracycline, ceftazidime, ciprofloxacin, ampicillin, and ampicillin/sulbactam, whereas the *S*. Typhimurium MDR strains mainly exhibited resistance to trimethoprim/sulfamethoxazole, tetracycline, ampicillin, and ampicillin/sulbactam. Additionally, nine *S*. Enteritidis isolates belonging to ST11 (7.8%)*,* five *S*. London strains belonging to ST155 (4.4%), four *S*. Rissen strains belonging to ST469, three *S*. Stanley belonging to ST29, and one strain each from the serovars of *S*. Derby and *S*. Goldcoast were also identified as MDR. Most *S*. Enteritidis MDR isolates were resistant to tetracycline, ampicillin, and ampicillin/sulbactam. In contrast, the *S*. London and *S*. Rissen isolates were primarily resistant to trimethoprim/sulfamethoxazole, tetracycline, ciprofloxacin, ampicillin, ampicillin/sulbactam, and trimethoprim/sulfamethoxazole, tetracycline, azithromycin, ampicillin, and ampicillin/sulbactam, respectively. Interestingly, the *Salmonella* diarizonae strain (22SAL094), a serovar rarely isolated from humans and warm-blood animals, was susceptible to all 14 antibiotics tested (File S2).

**TABLE 1:** Antimicrobial phenotype of these 384 *Salmonella* isolates

	Number of isolates^*a*^		
	S (%)	I (%)	R (%)
Trimethoprim/sulfamethoxazole	236 (61.5)	–^*b*^	148 (38.5)
Colistin	384 (100.0)	–	0 (0.0)
Ertapenem	380 (99.0)	2 (0.5)	2 (0.5)
Meropenem	382 (99.5)	0 (0.0)	2 (0.5)
Cefotaxime	320 (83.3)	1 (0.3)	63 (16.4)
Ceftazidime	315 (82.0)	9 (2.4)	60 (15.6)
Ceftazidime/avibactam	380 (99.0)	–	4 (1.0)
Tetracycline	137 (35.7)	6 (1.6)	241 (62.7)
Tigecycline	365 (95.1)	13(3.3)	6 (1.6)
Ciprofloxacin	150 (39.1)	166 (43.2)	68 (17.7)
Azithromycin	362 (94.3)	–	22 (5.7)
Amikacin	358 (93.2)	1 (0.3)	25 (6.5)
Ampicillin	138 (35.9)	1 (0.3)	245 (63.8)
Ampicillin/sulbactam	171 (44.5)	64 (16.7)	149 (38.8)

^*a*^ S, susceptible; I, intermediate; R, resistant.

^*b*^ –, not applicable.

**Fig 3:**
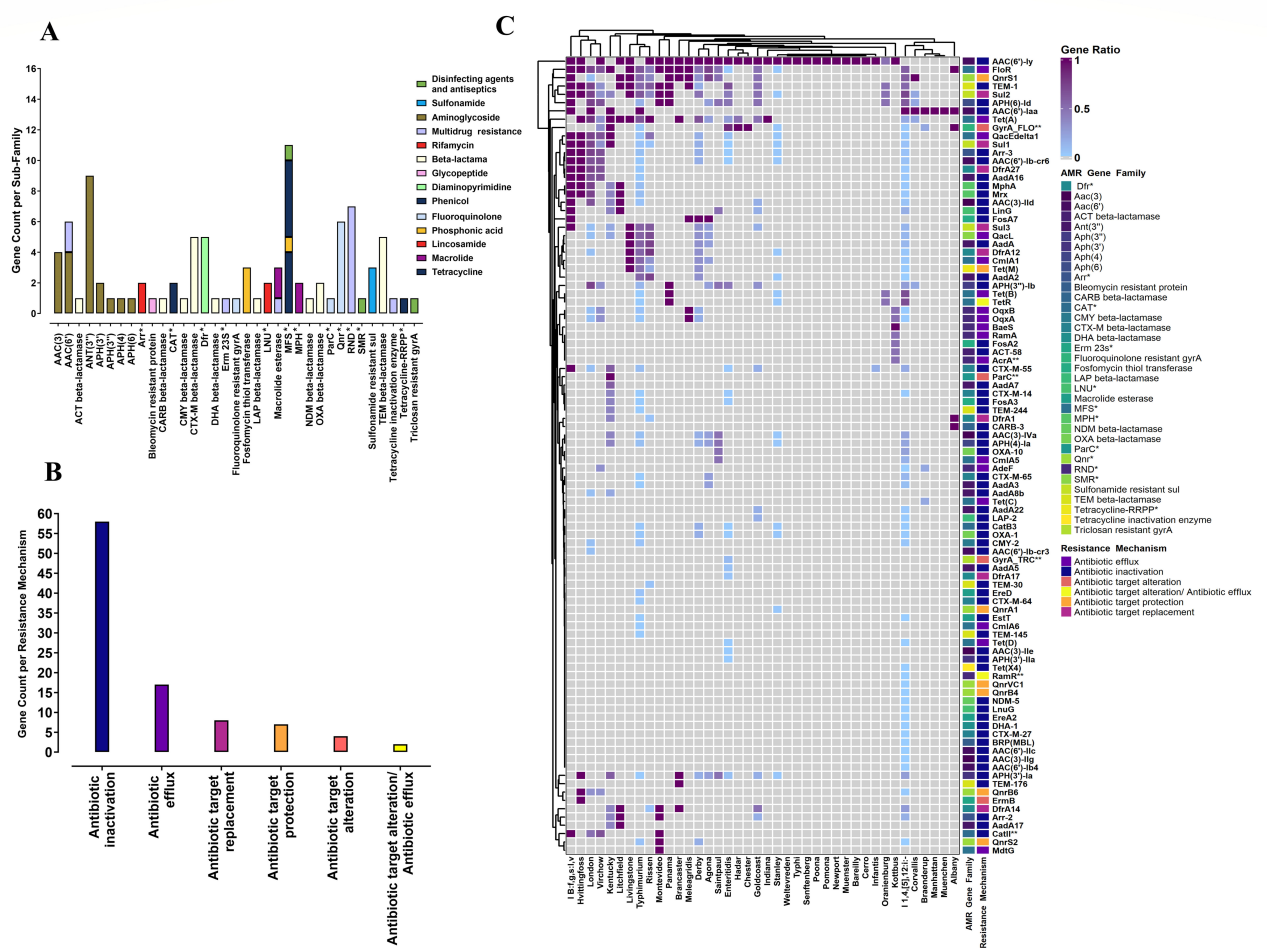
ARGs identified across 384 *Salmonella* isolates collected during 2017–2024. (**A**) A total of 96 AMR genes were identified across the 384 isolates and grouped into 35 subfamilies based on genotype patterns and function cluster analysis. Among these, five subfamilies contained more than five AMR genes. (**B**) 93 AMR genes were categorized into five groups according to their resistance mechanism. The majority of drug resistance was attributed to the inactivation of drugs by the corresponding AMR genes. (**C**) A heatmap illustrates the distribution of 96 AMR genes across the 40 serovars of *Salmonella* isolates. Gray areas in the heatmap indicate the absence of specific AMR genes in certain *Salmonella* serovars, whereas purple shades with varying intensities represent the frequency of a particular AMR gene detected in a specific serovar. AMR genes marked with the ** denote that target mutations caused the resistance. The AMR gene families labeled with * represent the abbreviated names. Dfr*—trimethoprim-resistant dihydrofolate reductase; Arr*—rifampin ADP-ribosyltransferase; CAT*—chloramphenicol acetyltransferase; Erm 23S*—23S ribosomal RNA methyltransferase; LNU*—lincosamide nucleotidyltransferase; MFS*—major facilitator superfamily antibiotic efflux pump; MPH*—macrolide phosphotransferase; ParC*—*Escherichia coli* ParC conferring resistance to fluoroquinolones; Qnr*—quinolone resistance protein; RND*—resistance-nodulation-cell division antibiotic efflux pump; SMR*—small multidrug resistance antibiotic efflux pump; Tetracycline-RRPP*—tetracycline-resistant ribosomal protection protein.

A total of 96 ARGs were identified among 384 *S*. *enterica* isolates, conferring resistance to 13 antimicrobial categories via six different resistance mechanisms (Fig. 3A and B). These 96 AMR genes were further classified into 35 subfamilies based on genotype patterns and function cluster analysis, with five subfamilies containing more than five AMR genes each (Fig. 3A). The 10 most prevalent ARGs were *aac(6*′*)-Iaa*(59%), *sul2*(52%), *bla_TEM-1_*(49.1%), *floR*(43.3%), *aac(6')-Iy* (40.5%), *APH(6)-I d*(40.2%), *qnrS1* (37.9%), *tet*(A) (32.9%), *tet*(R) (27.4%), and *tet*(B) (27.2%), which mediated resistance to six categories of antimicrobial agents, including the aminoglycoside, sulfonamide, beta-lactam, phenicol, fluoroquinolone, and tetracycline (Fig. 3C; File S3).

Intriguingly, 18 distinct β-lactam resistance genes from nine families, including *bla*_TEM_, *bla*_CTX-M_, *bla*_OXA_, *bla*_CMY_, *bla*_LAP_, *bla*_NDM_, *bla*_CARB_, *bla*_DHA_, and *bla*_ACT_, were also identified among these 384 *S*. *enterica* strains. The most prevalent β-lactam resistance genes were TEM-1 (49%, *n* = 188), CTX-M-55 (11.4%, *n* = 41), OXA-10 (9.1%, *n* = 35), CTX-M-65 (5.2%, *n* = 20), and CMY-2 (2.6%, *n* = 10). Further analysis of the distribution of ARGs across 40 serovars showed that *S*. I 1,4,[5],12:i:- harbored the highest number of ARGs (*n* = 64), followed by *S*. Typhimurium (*n* = 47), *S*. London (*n* = 28), and *S*. Enteritidis (*n* = 24) (File S3).

**Fig 4:**
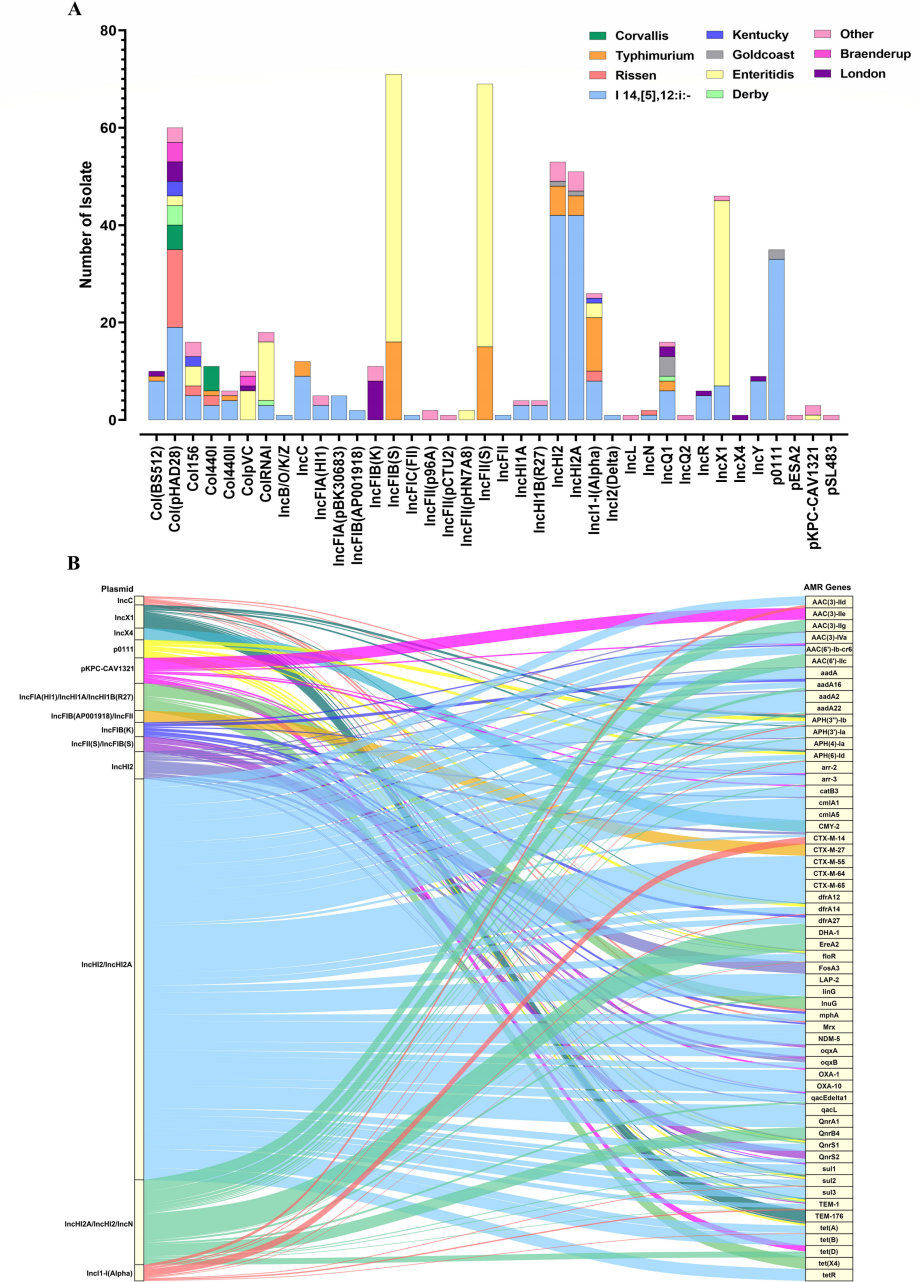
Prevalence and distribution of plasmids among the isolated *Salmonella* serovars. (**A**) Distributions of 39 identified plasmid replicons among 384 Salmonella isolates. Colors within each column represent the distribution of specific serovars. (**B**) Different plasmid types were associated with distinct patterns of ARGs among *Salmonella* isolates.

Transmissible plasmids serve as the primary vectors for the dissemination of antibiotic resistance genes among *Enterobacteria*. In this study, 39 distinct plasmid replicons were identified across 384 *S*. *enterica* isolates, with 275 strains harboring at least one plasmid. Notably, three *S*. Enteritidis isolates harbored five distinct plasmid replicons. Additionally, 16 isolates, comprising 11 *S*. Enteritidis, 3 *S*. Typhimurium, and one each of *S*. I 1,4,[5],12: i:- and *S*. London carried four distinct plasmid replicons (File S4). Additionally, 63 isolates carried three plasmid replicons, while 114 isolates harbored two plasmids. The most frequently detected plasmid types were *IncFIB(S)* (18.5%), followed by *IncFII(S)* (18.0%), *Col(pHAD28*) (15.6%), *IncHI2* (13.8%), *IncHI2A* (13.3%), and *IncX1* (12.0%) (Fig. 4A). The *IncFIB(S*) and *IncFII(S*) plasmids were exclusively associated with the serovars of *S*. Enteritidis and *S*. Typhimurium, with *S*. Enteritidis accounting for approximately 77% of these occurrences. In contrast, the *Col(pHAD28*) plasmid exhibited a broader distribution across 11 serovars, although *S*. I 1,4,[5],12:i:- and *S*. risen were the predominant serovars. Similarly, the *IncHI2* and *IncHI2A* plasmids were detected in seven distinct serovars; however, approximately 90% of these plasmids (48/53 and 46/51, respectively) were identified in *S*. I 1,4,[5],12:i:- and *S*. Typhimurium isolates. Although *S*. Enteritidis, *S*. I 1,4,[5],12:i:- and *S*. Typhimurium were the predominant serovars for most of the commonly detected plasmids, certain uncommon plasmids were identified in specific serovars. For instance, 5 of the 11 strains carrying the *Col440I* plasmid belonged to *S*. Corvallis, and 8 of the 11 isolates harboring the *IncFIB(K*) plasmid were associated with *S*. London. The *pKPC-CAV1321* plasmid was detected in only three isolates, two of which were from *S*. Virchow. Additionally, the plasmids *IncFII(pCTU2*), *IncL*, *IncQ2*, *IncX4*, and *pESA2* were identified in single isolates from *S*. Manhattan, *S*. Hadar, *S*. Montevideo, *S*. London, and *S*. Manhattan, respectively. The comprehensive plasmid distribution profiles across these 384 isolates are provided in File S4.

To further investigate the associations between the critical ARGs and plasmids, 40 isolates were subjected to long-read sequencing to obtain complete genome information for both the chromosome and plasmids. Hybrid plasmids such as *IncHI2/IncHI2A*, *IncFII(S)/IncFIB(S*), *IncFIA(HI1)/IncHI1A/IncHI1B(R27*), *IncFIB(AP001918)/IncFII*, and *IncHI2A/IncHI2/IncN* were frequently identified in these *Salmonella* isolates (Fig. 4B). Among these plasmids, the IncHI2/IncHI2A plasmid exhibited the highest diversity of resistance genes, including floR, tet(A), sul1/2/3, APH(3')-Ia, AAC(3)-Iva, aadA/A2/A6/A22, QnrS1, catB3, dfrA12/14/27, linG, mphA, cmlA1/A5, TEM-1, CTX-M-55/64/65, LAP-2, OXA-1/10, and NDM-5. In contrast, CTX-M-14 was primarily associated with the IncI1-I(Alpha) plasmid. CMY-2 was linked to the IncX4 plasmid, DHA-1 to IncHI2A/IncHI2/IncN, TEM-176 to IncX1, and FosA3 to IncHI2, respectively (Fig. 4B).

**Fig 5:**
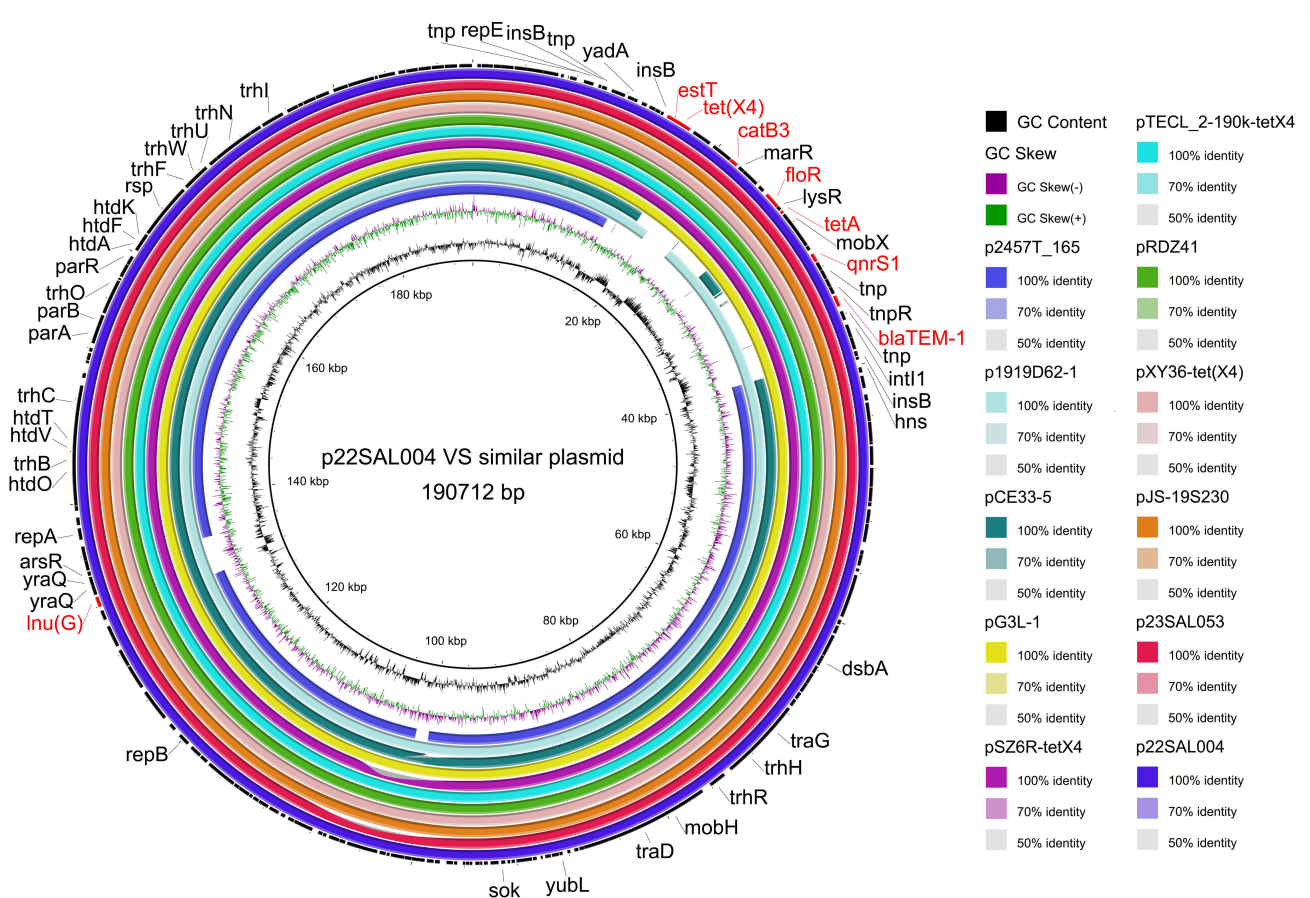
Sequence comparison of the *tet*(X4)-containing plasmid from *Salmonella* isolates 22SAL004 and 23SAL053 with similar plasmids identified in *Escherichia coli*, *Klebsiella pneumoniae*, and other species globally using BRIG. The GC content and GC skew of p22SAL004 are shown in the inner rings. Gene annotations for plasmid p22SAL004 are depicted on the outside black ring, with resistance genes highlighted in red.

Tigecycline is a last-resort antibiotic for treating serious infections caused by MDR pathogens. Notably, the *tet(X4*) gene was identified in two *S*. I 1,4,[5],12:i:- isolates (22SAL004 and 23SAL053), located on an identical IncFIA(HI1)-IncHI1A-IncHI1B(R27) hybrid plasmid. BLAST analysis of p22SAL004 revealed high similarity to plasmids previously reported in *Escherichia coli* (p1919D62-1 and Pce33-5), *Morganella morganii* (Pxy36-tetX4), *Enterobacter cloacae* (pTECL_2_190k_tetX4), *Salmonella enterica* (pJS19S230), *Leclercia sp*. (pG3L-1), *Citrobacter sp*. (pZS6R-*tetX4*), and *Klebsiella pneumoniae* (pRDZ41), all isolated in China, as well as in a *Shigella flexneri* strain (pXY36-*tetX4*) isolated from Japan. A further inspection of the genetic context revealed that the *tet(X4*) gene was associated with the macrolide hydrolase EstT, flanked by an IS1-family transposase and an IS91-like element ISVsa3-family transposase (Fig. 5).

### Virulence factor profiling and phylogenomic analysis of *Salmonella* isolates

The virulence genes present in the 384 *S*. *enterica* strains were analyzed using the VFDB database. The analysis identified 231 virulence genes across these isolates, which were classified into 10 categories. The number of virulence genes per isolate ranged from 95 to 175 (File S5). Of the 231 virulence genes, 127 were identified in more than 90% of the isolates. Among them, *csgA*, *csgB*, *csgE*, *csgF*, and *csgG* (involved in curli production and assembly), *fur* (a ferric iron uptake regulator), *phoP* (a global regulatory gene), and several type III secretion system genes—including *invB*, *invE*, *invF*, *invI*, *ssaD*, *ssaE*, *ssaK*, *ssaM*, and *ssaS* were detected in all 384 isolates. Interestingly, 21 of the 384 isolates were found to carry the *cdtB* gene, which encodes the typhoid toxin. These included six isolates of *S*. Goldcoast; two each of *S*. Typhi, *S*. Oranienburg, *S*. Poona, *S*. Chester, and *S*. Panama; and one each of *S*. Pomona, *S*. Diarizonae, *S*. Indiana, *S*. Muenster, and *S*. Montevideo. Among them, three isolates were recovered from environmental sources, including two *S*. Chester strains from poultry meat processing factories and one *S*. Goldcoast strain from hospital sewage. Additionally, the *east1* gene, which codes for a heat-stable enterotoxin homologous to the heat-stable enterotoxin *Sta* in Enterotoxigenic *Escherichia coli*, was detected in seven *S*. London isolates. Of these, six were recovered from human sources, while one strain (24CF1004) was isolated from hospital sewage.

**Fig 6:**
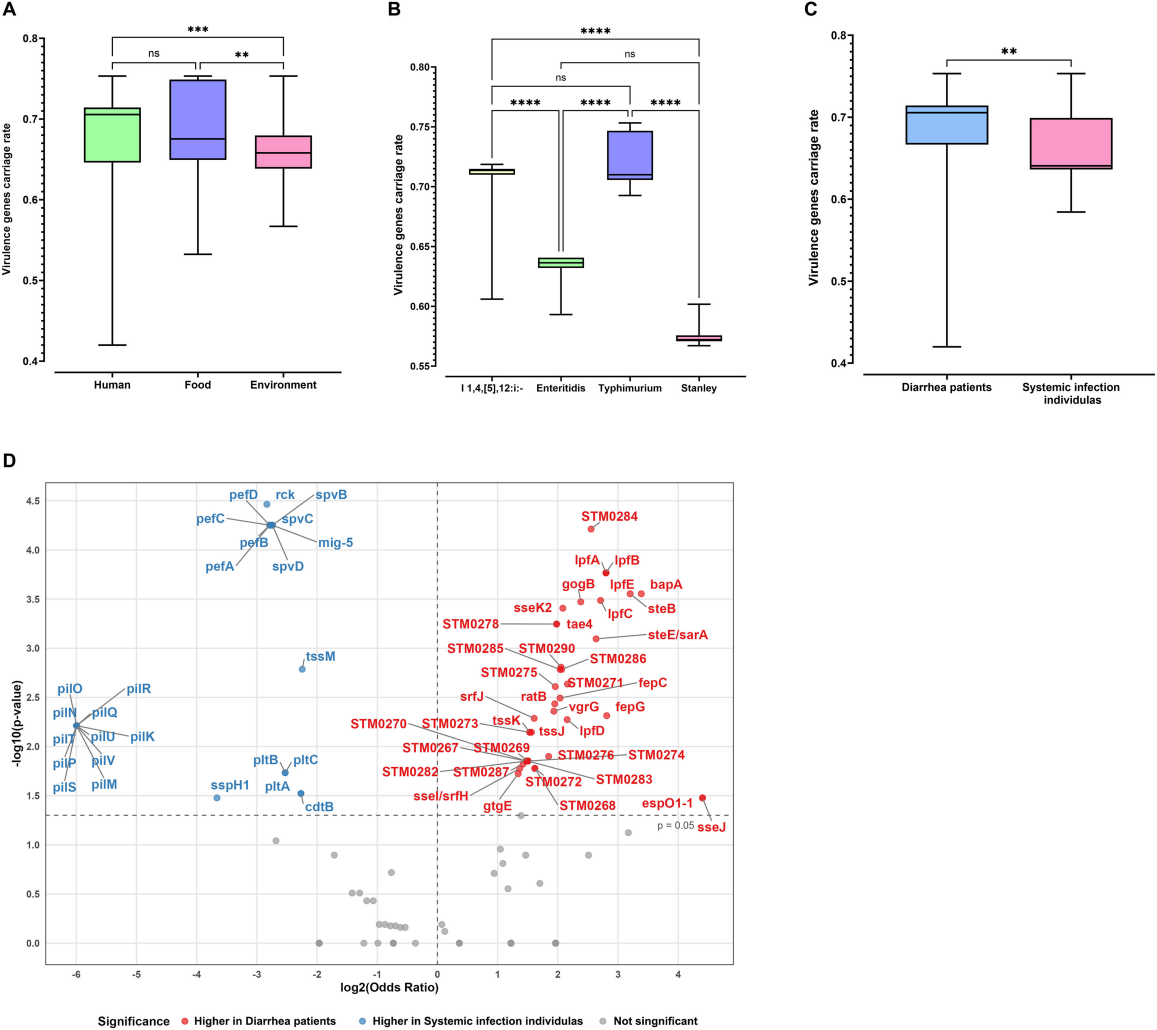
Dynamics and distributions of virulence genes across different sources and serovar strains. The virulence gene carriage rate for each isolate was calculated by dividing the number of virulence genes identified in that isolate by the total of 231 virulence genes detected across 384 isolates. (**A**) Distribution of virulence gene carriage rates among isolates from human, food, and environmental sources. (**B**) Distribution of virulence gene carriage rates in the four most frequently isolated serovars. (**C**) Distribution of virulence gene carriage rates in isolates from patients with diarrhea and those with systemic infection. (**D**) Volcano plot of differentially distributed virulence genes between isolates from diarrhea patients and systemic infection patients. *P* values: **P < 0.01; ***P < 0.001; ****P < 0.0001; ns. non signiﬁcant.

Interestingly, we also found that the distribution of virulence genes varied among different sources and serovars. The median carriage rates of virulence genes in strains isolated from humans (0.71) and food samples (0.68) were slightly higher than those from environmental sources (0.67) (Fig. 6A). Among the four most commonly isolated serovars, *S*. I 1,4,[5],12:i:- and *S*. Typhimurium strains harbored a significantly greater number of virulence genes compared to the *S*. Enteritidis and *S*. Stanley, with median carriage rates of 0.72, 0.71, 0.64, and 0.57, respectively (Fig. 6B). Furthermore, among strains isolated from humans, the median virulence gene carriage rate in isolates from diarrhea patients (0.71) was significantly higher than that observed in isolates from patients with systemic infections (0.64) (Fig. 6C). An in-depth analysis of these virulence genes with distinct distribution rates revealed that *gogB*, *lpfA*, *pfB*, *lpfC*, *lpfE*, *steE*, *sarA*, *sseK2*, *bapA*, and *STM0284* were more frequently detected in isolates from diarrhea patients (Fig. 6D). In contrast, the carriage rate of virulence genes *mig-5*, *pefA*, pefB, *pefC*, *pefD*, *spvB*, *spvC*, *spvD*, and *rck* reached 48% in strains from patients with systemic infection, which is considerably higher than that in strains isolated from diarrhea patients (11.4%–12.0%). Detailed information regarding the virulence gene of these 384 isolates is available in File S5.

**Fig 7:**
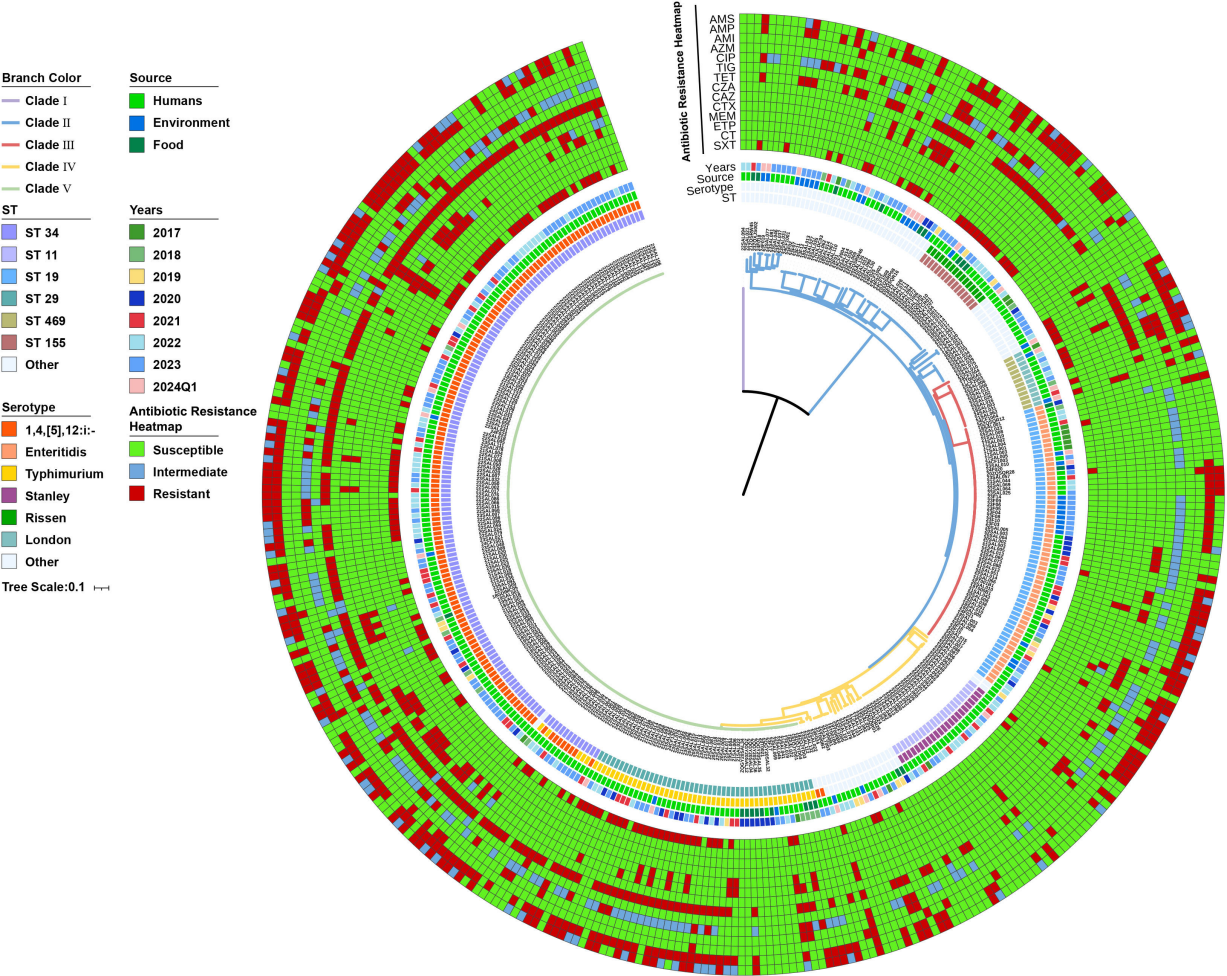
Phylogenetic analysis of 384 *Salmonella* isolates collected from varied sources during 2017–2024 in Zhaoqing, China. A maximum-likelihood tree was constructed using FastTree based on SNP alignments generated by SKA v1.0. The tree was further visualized and edited using the online tool iTOL, incorporating metadata such as serovar, ST types, source, collection time, and an AMR heatmap. Only ST types and serovars with more than 10 isolates are presented in detail; those with fewer than 10 isolates are collectively categorized as "other."

A maximum likelihood phylogenetic tree was constructed based on SNP alignments using FastTree to elucidate the relationships among these 384 isolates. The phylogenetic analysis revealed that the 384 *S*. *enterica* strains could be divided into five distinct clades: Clade I, Clade II, Clade III, Clade IV, and Clade V (Fig. 7). The isolate 22SAL094, identified as *S*. enterica subsp. diarizonae, was distinctly separated from all other isolates, clustering exclusively in Clade I. In contrast, Clade V represented the largest clade, comprising 196 strains, including 142 out of 144 of *S*. I 1,4,[5],12:i:- isolates, one strain of *S*. Oranienburg, and all 53 *S*. Typhimurium strains. The remaining two *S*. I 1,4,[5],12:I isolates, 23SAL112 and 23SAL132, were clustered into Clade IV. Clade IV consisted of 44 isolates, including all 23 *S*. Tanley and 5 *S*. Braenderup strains. *S*. Enteritidis, another prevalent serotype, was clustered exclusively in Clade III, which showed close genetic relatedness to *S*. London. Clade II predominantly clustered *S*. Rissen strains along with other serovar isolates. Notably, the 21 isolates that harbor the *cdtB* gene associated with typhoid toxin were clustered into Clade II.

**Fig 8:**
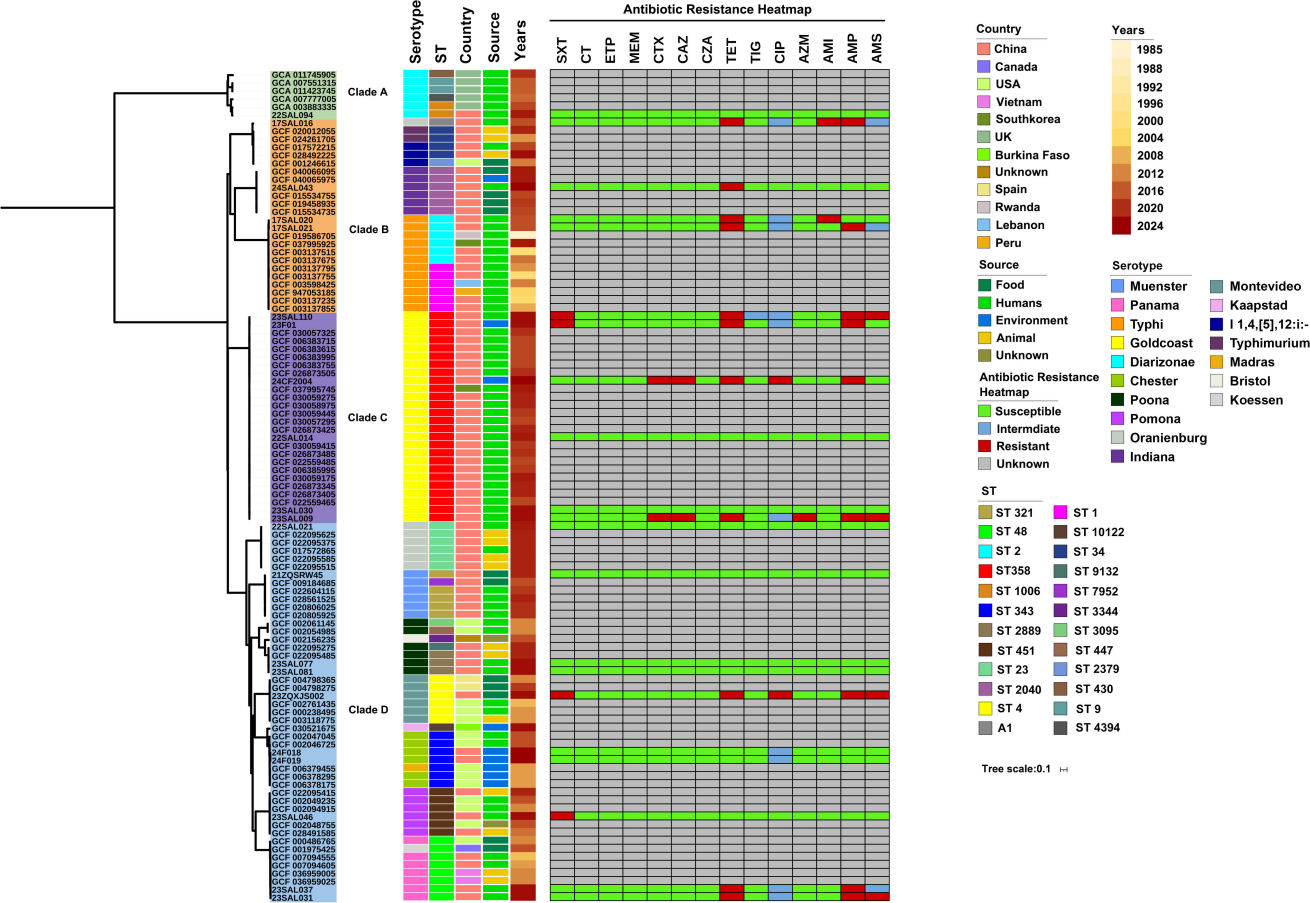
Phylogenetic analysis of the 21 *S*. *enterica* isolates harboring the typhoid toxin *cdtB* genes, along with 82 *S*. *enterica* strains of similar genomic compositions identified in the NCBI pathogen database. The maximum likelihood tree was built using FastTree based on SNP alignments generated by SKA v1.0, and then further visualized and edited by iTOL. Due to the absence of antibiotic susceptibility data for strains retrieved from the NCBI pathogen database, their drug susceptibility was marked as unknown in the Antibiotic Resistance heatmap.

To further investigate the genetic relationships between the 21 isolates harboring the *cdtB* genes and other *S. enterica* strains with similar genomic compositions identified in the NCBI pathogen database, a phylogenetic tree was constructed using the FastTree based on SNPs alignment (Fig. 8). The 103 *S*. *enterica* strains, including the 21 isolates carrying the *cdtB* genes, formed four distinct clades, designated as clades A to D (Fig. 8). Clade A, which is phylogenetically distinct from the other three clades, consists of six *S*. diarizonae strains, including one isolate (22SAL094) from this study, while the remaining five *S*. diarizonae were isolated over the past decade in the UK. Interestingly, all 26 *S*. Goldcoast strains, including six isolates harboring the *cdtB* genes collected in Zhaoqing, were exclusively clustered within Clade C. Among these 26 *S*. Goldcoast strains, only one was isolated from South Korea, while the others were all isolated from China. In contrast to Clade A, which contained a single serovar, Clades B, C, and D comprised multiple serovars, reflecting a mixed clustering pattern. The two *S*. Typhi strains isolated from humans in this study, although clustered with other *S*. Typhi strains in Clade B, exhibited the closest genetic relationship with an *S*. Typhi strain isolated from a human in Rwanda in 1985 (Fig. 8). Additionally, the other 10 isolates harboring the *cdtB* genes from Zhaoqing in this study, including those identified as *S*. Oranienburg, *S*. Muenster, *S*. Poona, *S*. Montevideo, *S*. Chester, *S*. Pomona, and *S*. Panama, were grouped within Clade D, which appears to be the most diversified Clade (Fig. 8).

## DISCUSSION

*Salmonella enterica* is a leading cause of foodborne infections, contributing significantly to the global disease burden, including diarrhea and severe multi-organ invasive diseases (25, 38). In China, an estimated 70%–80% of FBD outbreaks are attributed to *Salmonella enterica*, posing considerable public health challenges (5, 22, 23). To the best of our knowledge, this is the first comprehensive study to investigate the dynamics of clinical and non-clinical *Salmonella enterica* isolates within the tropical mountainous regions of China. Here, we systematically characterized the serovar distribution, MLST patterns, antimicrobial susceptibility profiles, resistance genes, plasmid replicons, virulence factors, and phylogenetic relationships of 384 *Salmonella enterica* isolates collected from humans, food, and the environment between 2017 and 2024 in Zhaoqing, Guangdong, a transitional region linking the southern coastal zone with inland China.

A total of 41 serovars were identified among the 384 isolates, indicating a diverse genetic background for the *Salmonella enterica* strains in this region. In Zhaoqing, the most prevalent serovars isolated from humans were *S*. I 1,4,[5],12:i:- (43.5%), *S*. Enteritidis (14.2%), *S*. Typhimurium (13%), and *S*. Stanley (7.2%). This predominance of *S*. I 1,4,[5],12:i:- aligns with the data of the whole province, where *S*. I 1,4,[5],12:i: accounted for 35.6% (*n* = 5,372) of 15,091 isolates collected by the Guangdong Provincial CDC from 2009 to 2019 (27). By contrast, in Hangzhou, Zhejiang Province, *S*. Enteritidis was reported as the most prevalent serovar, while nationally *S*. Typhimurium remains the most commonly isolated serovar, indicating substantial regional variation in human *Salmonella* infections across China (25, 26). We also observed distinct serovar distribution patterns across sources. Unlike the human isolates, where *S*. I 1,4,[5],12:i:- (43.5%) dominated, the most frequently isolated serovars from the environment and food samples were *S*. Enteritidis (35.1%), *S*. Corvallis (13.5%), or *S*. Typhimurium (39.1%), and *S*. Rissen (13.0%), respectively. The high prevalence of *S*. Enteritidis in poultry farms and meat processing factories aligns with the recent One Health meta-analysis by Chen, J., et al., which identified *S*. Enteritidis and *S*. Indiana as the dominant serovars in poultry, serving as critical vehicles for S. Enteritidis transmission nationwide (39). Approximately 10% of NTS cause severe extraintestinal complications such as endovascular foci, visceral abscesses, and bacteremia (38, 40). In our study, the *S*. Enteritidis (40%), *S*. Derby (12%), *S*. Typhimurium (12%), and *S*. Typhi (8%) were the predominant serovars associated with human systemic infections. These findings align with recent research by Zhou et al., which found that *S*. Enteritidis was the most prevalent serovar, accounting for 43.3% of 1,115 iNTS isolates collected in China from 1993 to 2023 (38). Forty-seven distinct ST types were identified across the 384 isolates, with ST34 (comprising 142 strains of *S*. I 1,4,[5],12:i:-and five strains of *S*. Typhimurium), ST11 (61 *S*. Enteritidis isolates), and ST19 (48 *S*. Typhimurium isolates) as the largest three ST groups. *S*. Typhimurium has been reported to possess a higher diversity of sequence types compared to other serovars, and ST34 from pig and ST19 from chicken origin of *S*. Typhimurium were documented to be mainly associated with child and adult gastro-infection, respectively, at a national level (26). However, no similar host preference was observed for ST34 and ST19 isolates in our study. Additionally, this analysis did not detect ST313 *S*. Typhimurium, a virulent lineage prevalent in various African countries responsible for invasive human infections (40, 41).

The diverse genetic background of *Salmonella enterica* strains in this region, as evidenced by the highly distinct serovars and ST types identified, indicates a more complex AMR profile. We identified 96 ARGs among these 384 isolates, conferring resistance to 13 categories of antimicrobial agents through six different resistance mechanisms. Notably, high resistance rates were observed for ampicillin (63.8%), tetracycline (62.7%), ampicillin/sulbactam (38.8%), and trimethoprim/sulfamethoxazole (38.5%) (Table 1). High levels of ampicillin resistance have also been documented in Shanghai, Zhejiang, and Jiangsu provinces. In contrast, ampicillin resistance rates in geographically isolated countries, such as Australia and the United Kingdom, were only 9.9% and 17.3%, respectively (42–44). In agreement with the above high AMR rates, a significant prevalence of beta-lactam ARG *bla*_TEM-1_ (49.1%), sulfonamide ARGs *sul2* (52%), *sul3* (17.8%), tetracycline ARGs *tet*(A) (32.9%), *tet*(R) (27.4%), *tet*(B) (27.2%), and trimethoprim ARGs *dfrA12* (14.1%), *dfrA14* (12.5%), and *dfrA27* (5.2) were correspondingly detected among these 384 isolates. Ciprofloxacin, cefotaxime, and ceftazidime are clinically important for treating salmonellosis (45). Alarmingly, resistance rates reached 17.7% for ciprofloxacin, 16.4% for cefotaxime, and 15.6% for ceftazidime, raising concerns about the potential for treatment failure when fluoroquinolones and third-generation cephalosporins are empirically prescribed in this tropical mountainous region. Tigecycline is a last-resort therapeutic option for complicated infections caused by MDR pathogens (46). In this study, six strains—four belonging to *S*. I 1,4,[5],12:i:-, one each for *S*. Typhimurium and *S*. Stanley—all isolated from children under three years of age, exhibited tigecycline resistance. However, only two *S*. I 1,4,[5],12:i:- strains harbored the *tet*(X4) gene, which is linked to the macrolide hydrolase *EstT* located on an IncFIA(HI1)- IncHI1A- IncHI1B(R27) hybrid plasmid. Although similar plasmids have been identified in different species isolated in China, including *Escherichia coli* (p1919D62-1 and Pce33-5), *Enterobacter cloacae* (pTECL_2_190k_tetX4), *Morganella morganii* (Pxy36-tet(X4), *Enterobacter cloacae* (pTECL_2_190k_tetX4), *Salmonella enterica* (pJS19S230,pSM2301-tetX4, and pT50–1-tetX), and *Klebsiella pneumoniae* (pRDZ41), this is the first report of *tet*(X4) in *S*. I 1,4,[5],12:i:- strain isolated from an 8-month-old infant in South China (47, 48). Aside from the *tet*(X) variants responsible for tigecycline resistance, the RND efflux pump *tmexCD-toprJ* has also been implicated in tigecycline resistance (49, 50). Interestingly, the *toprJ* gene was undetected in any of the six strains resistant to tigecycline, including the four strains lacking the *tet*(X4) gene. Future research should thoroughly investigate the mechanism underlying tigecycline resistance in these strains.

Self-transmissible plasmids carrying diverse transposons and integrons, associated with virulence factors and ARGs, are pivotal for the adaptation and evolution of *Enterobacteriaceae* species (51). In this study, we identified 39 distinct plasmid replicons among the 384 *S*. *enterica* strains, indicating a rich diversity of plasmids within *S. enterica* in this tropical mountainous region. A total of 275 out of 384 strains were found to contain at least one plasmid, with the most frequently identified replicons being *IncFIB* (S) (18.4%), *IncFII(S) (*17.9%), *Col(pHAD28*) (15.6%), *IncHI2* (13.8%), *IncHI2A* (13.2%), and *IncX1* (11.9%). This finding is in agreement with previous national-level observations indicating that *IncFII(S*), *IncFIB(S*), *Col(pHAD28*), *IncHI2,* and *IncX1* were among the most common plasmid replicons, with *IncFII(S*) and *IncFIB(S*) plasmids accounting for an even higher frequency, reaching 28.49% and 27.42% of the 1,962 isolates, respectively (26). Additionally, our study further demonstrates that the *IncHI2/IncHI2A* hybrid plasmid primarily contributes to the dissemination of *floR*, *tet(A), sul1/2/3, aph(3')-Ia, aac(3)-IVa, aadA/A2/A6/A22, qnrS1, catB3, dfrA12/14/27, lnu(G), mph(A), cmlA1/A5, TEM-1, CTX-M-55/64/65, LAP-2, OXA-1/10,* and *NDM-5* among Salmonella spp. In contrast, the dissemination of *CTX-M-14*, *CMY-2*, *DHA-1*, and *FosA3* was attributed to *IncI1-I(α*), *IncX4*, *IncHI2A/IncHI2/IncN*, and *IncHI2* plasmids, respectively.

We identified a total of 231 virulence genes across the 384 S. enterica strains, among which 127 virulence genes were detected in more than 90% of the isolates. However, a significant difference in the distribution of virulence genes was observed among the isolates from diarrheic patients and systemic infection patients. Genes encoding carbonic anhydrase (*mig-5*), plasmid-encoded fimbriae (*pefA*, *pefB*, *pefC*, and *pefD*), type III secretion system effectors (*spvB*, *spvC*, and *spvD*), and resistance to complement killing (*rck*) were significantly more prevalent in strains from patients with systemic infection than in those from diarrhea cases. This observation is in line with the function of the type III secretion system effectors (*spvB*, *spvC*, and *spvD*), which have been reported to aggravate systemic infections in mice via disrupting intestinal epithelial barrier integrity, inhibiting pyroptosis, and inducing intestinal inflammation (52–54). Similarly, the *rck* gene contributes to Salmonella’s invasion ability by conferring high resistance to complement-mediated bactericidal activity and facilitating Zipper-like internalization (55, 56). However, the roles of carbonic anhydrase (*mig-5*) and plasmid-encoded fimbriae (*pefA*, *pefB*, *pefC*, and *pefD*) in the course of *S. enterica* systemic infection remain unclear and deserve further investigation.

The typhoid toxin, a unique A2B5 exotoxin encoded by *CdtB*, *PltA,* and *PltB* genes, is a key virulence factor for *S*. Typhi (57, 58). Recent studies have reported the emergence of *cdtB* in several NTSs, such as *S*. Goldcoast and *S*. Indiana (41, 59, 60). In our investigation, 21 isolates harbored the *cdtB* gene, including six *S*. *Goldcoast* isolates, two each of *S*. Typhi, *S*. Oranienburg, *S*. Poona, *S*. Chester, and *S*. Panama. Phylogenetic analysis revealed that most of these 21 strains were genetically distinct from the *S*. Typhi strains and were distributed sporadically across four distinct clades (Fig. 7). Although most of these 21 strains clustered closely with strains from China, four isolates, namely 22SAL094 (*S*. diarizonae), 23ZQXJS002 (*S*. Montevideo), 24F018 and 24F019 (both *S*. Chester), exhibited closer relationships with strains isolated from the UK, Spain, and the United States, respectively, suggesting diverse dissemination of *cdtB*-containing *S. enterica* strains globally.

Phylogenetic analysis clustered the 384 *S*. *enterica* strains into five distinct clades, with Clade V comprising 142 out of 144 of *S*. I 1,4,[5],12:i:- isolates and all 53 *S*. Typhimurium strains. In contrast, the remaining two *S*. I 1,4,[5],12:I:- isolates, 23SAL112 and 23SAL132, exhibited different relationships with other *S*. I 1,4,[5],12:i:- and were grouped into Clade IV alongside *S*. Tanley and *S*. Braenderup strains. Epidemiological analysis revealed that these two strains were isolated from individuals recently relocated to Zhaoqing from another remote city, suggesting that they were likely imported rather than locally originated. This finding, enabled by WGS-based phylogenetics, also highlights the utility of genomics for tracing transmission dynamics and distinguishing endemic from imported strains.

### Conclusion

In conclusion, our study provides the first comprehensive characterization of the temporal and spatial dynamics of *Salmonella enterica* strains in the tropical mountainous regions of Southern China. We found that *S*. I 1,4,[5],12:I:- (ST34), *S*. Enteritidis (ST11), *S*. Typhimurium (ST19 and ST34), *S*. Stanley (ST29), *S*. Rissen (ST469), and *S*. London (ST155) were the predominant serovars in Zhaoqing. In contrast, systemic infections in kids were mainly caused by *S*. Enteritidis, S. Derby, and *S*. Typhimurium. High resistance rates to ampicillin, tetracycline, ampicillin/sulbactam, and trimethoprim/sulfamethoxazole were observed among these isolates, which were likely caused by the widespread presence of resistance genes such as *bla*_TEM-1_, *sul2*, *sul3*, *tet*(A), *tet*(R), *tet*(B), *dfrA12*, and *dfrA14*. Notably, the *tet*(X4) gene located on an IncFIA(HI1)- IncHI1A- IncHI1B(R27) hybrid plasmid was unexpectedly detected in two *S*. I 1,4,[5],12:I:- isolates. Additionally, the enterotoxin gene *east1* and the typhoid toxin gene *cdtB* were detected in several common serovars, besides *S*. Typhi. These findings address significant data gaps regarding dominant serovars and AMR profiles of *S. enterica* in the tropical mountainous regions of South China, thus providing valuable insights for clinical treatment and public health intervention strategies.

## Data Availability

Both the short-read sequencing data for 384 *Salmonella enterica* isolates and the long-read sequencing data for selected 40 isolates have been deposited in the National Genomics Data Center (NGDC) under project number PRJCA039050. The detailed genomic accession numbers for each isolate are provided in File S1.

## SUPPLEMENTAL MATERIAL

File S1 spectrum.02676-25-s0001.xls Epidemiological and molecular characteristics of 384 *Salmonella* isolates from Zhaoqing, China (2017-2024).

File S2 spectrum.02676-25-s0002.xls MIC values and antimicrobial resistance heatmap of 384 *Salmonella* strains.

File S3 spectrum.02676-25-s0003.xls Antimicrobial resistance gene predictions, distribution heatmap, and statistical data.

File S4 spectrum.02676-25-s0004.xls Plasmid replicon predictions and their distribution and associations with ARGs across different serovars.

File S5 spectrum.02676-25-s0005.xls Virulence gene predictions and presence in each isolate.

ASM does not own the copyrights to Supplemental Material that may be linked to, or accessed through, an article. The authors have granted ASM a non-exclusive, world-wide license to publish the Supplemental Material files. Please contact the corresponding author directly for reuse.
